# A Multifaceted Nurse- and Web-Based Intervention for Improving Adherence to Treatment in Patients With Cardiovascular Disease: Rationale and Design of the MIRROR Trial

**DOI:** 10.2196/resprot.5750

**Published:** 2016-09-13

**Authors:** Angelien Sieben, Hein AW van Onzenoort, Kees J van Laarhoven, Sebastian JH Bredie

**Affiliations:** ^1^ Radboud University Nijmegen Medical Centre Department of Surgery, Division of Vascular Surgery Radboud University Nijmegen Medical Centre Nijmegen Netherlands; ^2^ Radboud University Nijmegen Medical Centre Department of Pharmacy Radboud University Nijmegen Medical Centre Nijmegen Netherlands; ^3^ Maastricht University Medical Centre Department of Clinical Pharmacy and Toxicology Maastricht University Medical Centre Maastricht Netherlands; ^4^ Radboud University Nijmegen Medical Centre Department of Surgery, Division of Surgery Radboud University Nijmegen Medical Centre Nijmegen Netherlands; ^5^ Radboud University Medical Centre Department of General Internal Medicine Radboud University Medical Centre Nijmegen Netherlands

**Keywords:** medication adherence, cardiovascular, eHealth, nursing

## Abstract

**Background:**

Poor adherence to medication is one of the limitations in the treatment of cardiovascular diseases, thereby increasing the risk of premature death, hospital admissions, and related costs. There is a need for simple and easy-to-implement interventions that are based on patients’ perspectives, beliefs, and perceptions of their illness and medication.

**Objective:**

The objective is to test the effectivity of this intervention to improve medication adherence in patients with established cardiovascular disease, that is, in secondary prevention.

**Methods:**

In this study the effect of a personalized visualization of cardiovascular risk levels through a website aiming at supporting self management in combination with a group consultation and communication intervention by a nurse on adherence to treatment in 600 patients with manifest cardiovascular diseases will be assessed. The health belief model was chosen as main theoretical model for the intervention.

**Results:**

Primary outcome is adherence to treatment calculated by refill data. Secondary outcomes include the Beliefs about Medication Questionnaire and the Modified Morisky Scale. Patients are followed for one year. Results are expected by 2015.

**Conclusions:**

This study assesses adherence to treatment in a high-risk cardiovascular population by applying an intervention that addresses patients’ capacity and practical barriers as well as patients’ beliefs and perceptions of their illness and medication.

**ClinicalTrial:**

ClinicalTrials.gov NCT01449695; https://clinicaltrials.gov/ct2/show/NCT01449695 (Archived by WebCite at http://www.webcitation.org/6kCzkIKH3)

## Introduction

### Background and Rationale

According to the World Health Organization, almost 50% of all chronic patients do not adhere to their prescribed drug regimen [1]. This is also true for cardiovascular diseases (CVD); only 60% of all cardiovascular patients adhere to their cardiovascular medications (eg, statins, antihypertensives, antithrombotic agents) [2]. This prevalence is similar across all individual CVD medications and occurred in patient who take these medications for primary and secondary prevention of CVD [2]. These figures are startling given that poor adherence results in an increased risk of death in cardiovascular patients [3-5].

Current methods for improving adherence are mostly complex and have limited effectiveness; simple interventions that are easy to implement in daily practice are preferred [6]. Evidence suggests that interventions should be based on the patients’ perspective [7], target patients’ capacity and practical barriers, and address their beliefs and perceptions regarding illness and medication [8,9]. In CVD, life-long adherence is important, and interventions should improve patients’ intentions to take medication as well as solve emergent practical barriers.

These principles were used in the development of the current trial. Specifically, the intervention is based on the health belief model (HBM) [10,11], tailored for the specific purpose of this trial. HBM provides a useful framework for designing behavior change strategies [12]. It is based on the understanding that a person will take health‐related action (eg, being adherent to cardiovascular medication) given four main factors. The first two factors are perceived susceptibility and perceived severity: understanding of the high personal risk and seriousness of a condition (eg, because of the cardiovascular event in the past I am at greater risk for another cardiovascular event). The third factor is perceived benefit, or a belief that a negative health condition can be avoided (eg, being adherent to the cardiovascular medication can help to prevent another cardiovascular event). The last factor is perceived barriers. Cue to action and self‐efficacy and the belief in the ability to successfully undertake the recommended health action (eg, I know how to take my medication on a daily basis) [12,13].

### Trial Design and Aim of the Study

The study will use a single‐center, prospective, randomized controlled clinical trial design and examine the effectiveness of a new intervention that incorporates HBM and behavior change strategies to improve adherent behavior in cardiovascular patients. The intervention consists of a patient-based screening method, a specific nurse-based intervention (structural informative consulting and motivational counseling), and personalized visualization of cardiovascular risk levels via a website. The objective is to test the effectiveness of this intervention to improve medication adherence in patients with established CVD (ie, in secondary prevention).

## Methods

### Study Setting

Participants will be drawn from a hospital‐wide screening program. This screening program is situated at the cardiovascular outpatient clinics in an academic medical center in Nijmegen, the Netherlands. All new patients diagnosed in the last 6 months with acute coronary syndrome, peripheral arterial disease, an aneurysm of the aorta, or stroke/transient ischemic attack (TIA) and referred to the departments of vascular surgery, neurology, or cardiology are automatically included in this program.

### Eligibility Criteria

From this population, participants aged 18 years and older will be selected based on the following inclusion criteria: presence of CVD (acute coronary syndrome, peripheral arterial disease, an aneurysm of the aorta, or stroke/TIA), diagnosed in the last 6 months by a medical specialist, willingness to remain in follow‐up for a period of one year, and provision of signed informed consent. Exclusion criteria are pregnancy (reported by the patient), severe comorbidity (eg, a mental health diagnosis considered by a physician to be a contraindication), problems with the Dutch language (reported by the nurse), or logistic problems such as lack of computer access.

### Intervention

For the intervention, participants will be split in three groups. Participants in group I (control group) receive only usual care. Group II participants receive usual care plus access to a personalized website. For the group III participants, in addition to usual care and access to the personalized website, the intervention program will also include a single group consultation of 60 minutes led by a nurse and a pharmacist followed by two individual consultations of 30 minutes with a nurse.

We want to test if treatment II (only the Web portal) can give the same results as treatment III (the Web portal and the single group consultation followed by two individual consultations). The need for low-cost effective interventions in our health care system led to the motivation for this 3-arm protocol.

#### Usual Care (Groups I, II, and III)

All new CVD patients receive the hospital‐wide screening program according to the Dutch guidelines [14] (based on the European guidelines [15]). The screening assesses cardiovascular risk factors in all patients with CVD. It screens for lifestyle risk factors, blood lipid levels, blood pressure, waist circumference, body mass index, blood glucose levels, and a family history of CVDs. Lifestyle is evaluated through a questionnaire which is a compilation of existing validated questionnaires regarding demographic data, smoking, alcohol use, physical activity, and eating habits. For each of these lifestyle issues, the patient’s motivation to change is evaluated [16]. Adherence is measured by the Modified Morisky Scale (MMS) [17] and the Beliefs about Medication Questionnaire (BMQ) [18]. Medication use will be monitored. If necessary and if the patients agree they attend consultations with a nurse based on motivational interviewing to help them lose weight, stop using alcohol, or stop smoking.

According to European guidelines [15], all patients with established CVDs (this means all participants of this trial) should have antiplatelet therapy (eg, aspirin or clopidrogel) and a lipid lowering drug (eg, simvastatin or atorvastatin). The use of antihypertensive drugs is dependent on the systolic blood pressure. Except for the specific additions for the study, all participating and nonparticipating patients receive the same regular preventive cardiovascular care including monitoring of medication use. All patients receive regular vascular care from their medical specialist.

#### Website (Groups II and III)

The website contains an individualized Web portal called Interactive File Vascular Care (Interactive Dossier Vaatzorg, or iVAZ). This is developed to support patient-based self-evaluation and management [19,20]. Patients can log on and see their own cholesterol levels, blood pressure, and lifestyle (smoking habit, exercise, and eating habits) in a risk monitor. Patients can ask questions by email to their nurses, and they can enter changes in their medication. iVAZ provides risk communication, the feedback of clinical outcome will be provided individually, and patients are invited to be active in managing their illness and medication.

#### Group and Individual Consultations (Group III)

For group III, the intervention program will also include a single group consultation of 60 minutes led by a nurse and a pharmacist followed by two individual consultations of 30 minutes with a nurse.

During the group consultations patients receive information about their disease, cardiovascular medication (statins and antihypertensive and antithrombotic agents), and the importance of treatment adherence. Patients will receive an information booklet with all information presented during the plenary session. At the end of this consultation patients are asked to keep a diary of their medication intake during a 2-week period and to set a personal goal for the upcoming individual consultation with a nurse. The group consultation is regarded as an efficient way to increase knowledge and understanding of the risks. It also provides a gathering with other patients (peers).

During individual consultations, the intervention is further tailored based on the goal previously set, patient’s concerns, and necessities using the results of the screening questionnaire (see Data Collection). The following topics will be discussed during the individual consultation: patient's motivation and confidence (barriers, concerns, and positive self-motivational statements about their adherence behavior), options for increasing adherence to treatment, and a global summary of the counseling session.

Both the group and the individual consultations take place at the outpatient clinic. The involved nurses have had training in motivational interviewing [21] and were especially trained for this intervention by a psychologist.

**Figure 1:**
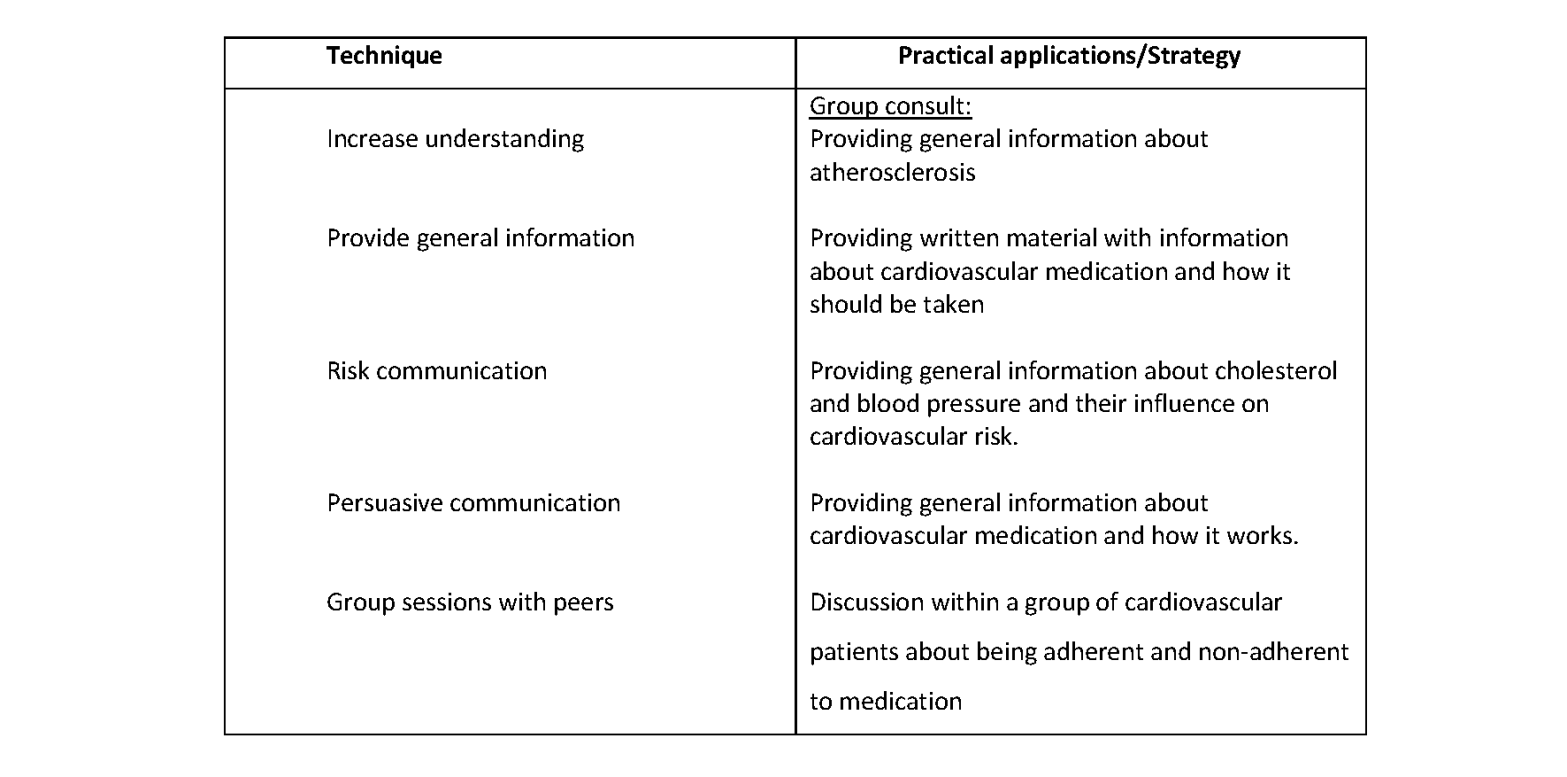
Techniques and applications influence perceived susceptibility in the current trial. The main determinant behind perceived susceptibility is a lack of knowledge regarding prescribed medications and the influence on risk reduction.

**Figure 2:**
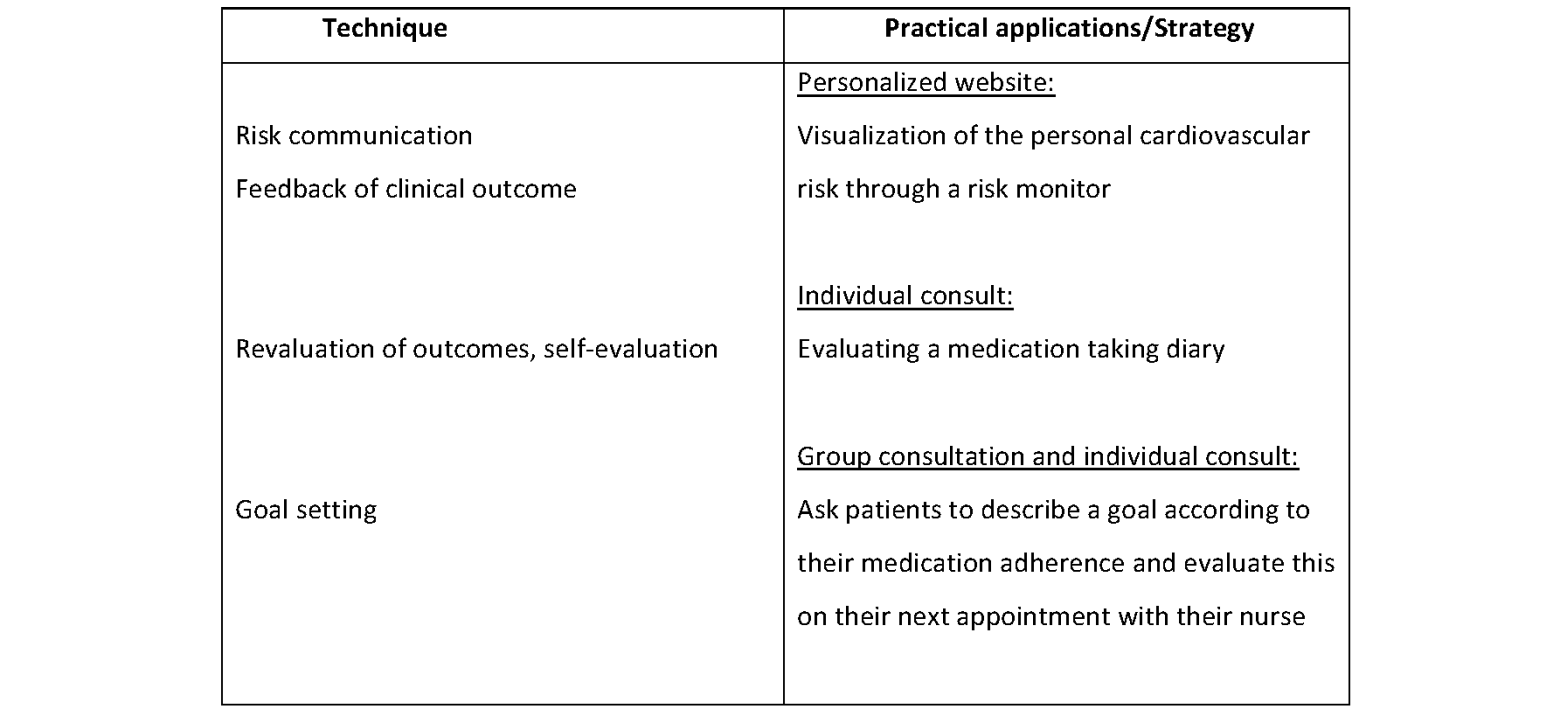
Techniques and applications influence perceived severity in the current trial. The main determinant behind perceived severity is patients’ beliefs, perception and management of their illness (awareness, outcome expectations).

**Figure 3:**
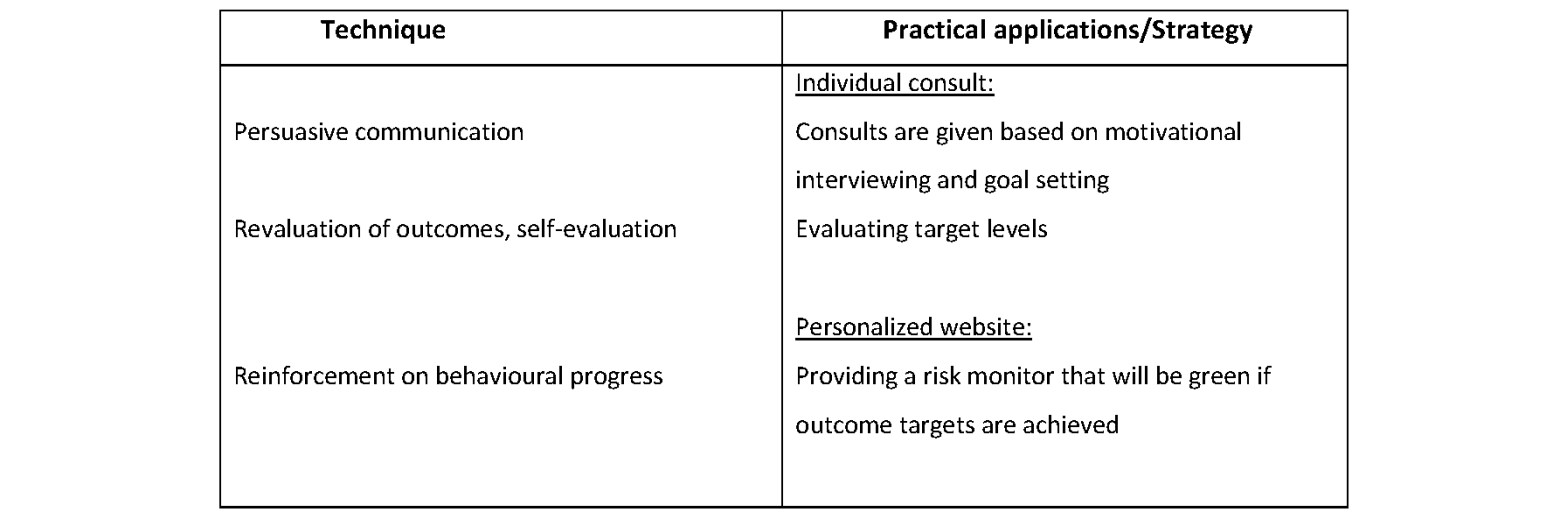
Techniques and applications influence perceived benefits in the current trial. The main determinant behind perceived benefits is patients' beliefs, perceptions, and management of their illness (awareness).

**Figure 4:**
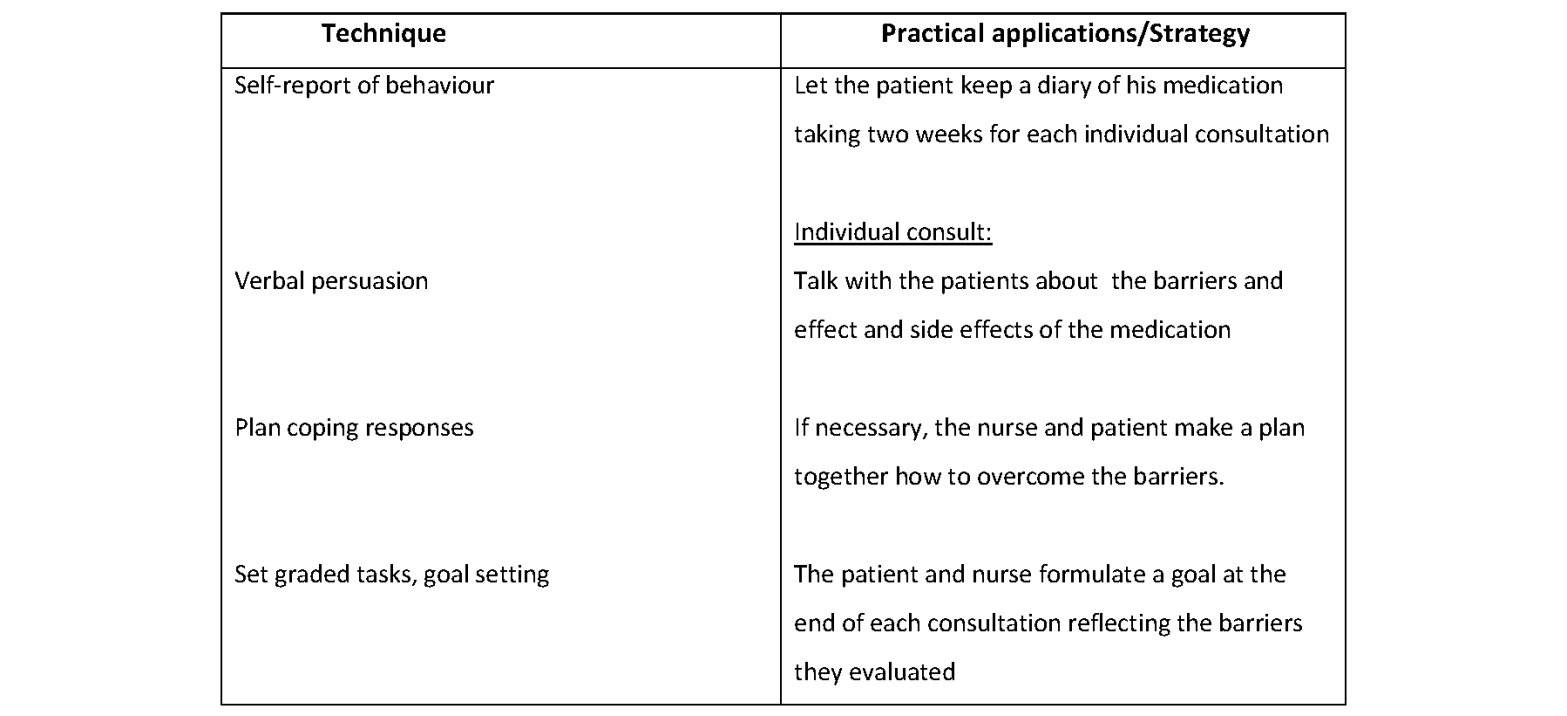
Techniques and applications influence perceived barriers, cue to action, and self-efficacy in the current trial. The main determinant behind perceived barriers is skills and self-efficacy.

For each of the constructs, we used the recommended behavior change strategies [12,13]. We tailored the intervention further by using the taxonomy of Abraham and Michie [22,23] and the coding manual by de Bruin [24] to categorize the behavior change techniques to be included in the intervention. For each of the components of HBM, the determinants, techniques, and application strategy were developed and are detailed in Figure 1-4.

## Results

### Primary Outcome

The primary outcome of our study is adherence to the CVD medication (classified by the Anatomic Therapeutic Chemical classification system) measured with a dedicated calculation of refill data of the used plated aggregation inhibitors and lipid modifying agents obtained from patient’s pharmacy.

Refill records of computerized pharmacy systems will be collected from 3 years prior to a patient’s cardiovascular event through up to 3 years after the study follow‐up period. Prescription records include the names of all of the dispensed drugs, prescribed daily dose, quantity dispensed at each pharmacy fill, and the dates of the prescription fills. Adherence will be calculated for the CVD medications as the theoretical duration divided by the period between the start date and the date of the last prescription filled. The theoretical duration will be calculated by dividing the number of units dispensed by the prescribed daily dose [25].

Patients with an adherence level of at least 80% will be classified as adherent, and patients with an adherence level less than 80% will be classified as nonadherent. Secondary prevention studies showed that patients with an adherence of less than 80% have an increased risk of death [26].

Refill adherence rates have been used extensively for the assessment of drug acquisition and dispensing. Compared with electronic monitoring, refill data provide researchers with a relatively simple method for investigating exposure to medication in large populations [27-29]. Moreover, this method is suitable for investigating long‐term persistence to treatment and gaps in medication supply [30].

### Secondary Outcomes

All secondary outcome measurements will be obtained just before inclusion (in the usual care screenings program) and one year after inclusion. The secondary outcome measurements include clinical responses to drug therapy (eg, cholesterol level), self-report questionnaires, and changes in systolic blood pressure.

Clinical responses to drug therapy will be recorded. A recorded low-density lipoprotein cholesterol level above 20% of preestimated low-density lipoprotein cholesterol reduction during follow-up will be considered as possible indication of poor adherence. If the patient also uses antihypertension drugs, the blood pressure on baseline will be compared to blood pressure after one year and will need to be within target blood pressure for cardiovascular risk management (systolic <135 mm Hg). These office blood pressure measurements are performed according to the recommendations of the European Society of Hypertension [15] with a validated automated device; data will be based on a mean of four office measurements.

Second, two validated self-report questionnaires will be used. The MMS will be used to measure adherence [17]. Each of the 8 items measures a specific medication‐taking behavior. MMS scores can range from 0 to 8 and can be classified into three levels of adherence: low adherence (score of less than 6), medium adherence (score of 6 to less than 8) and high adherence (score of 8) [31]. The BMQ will be used to provide information about the beliefs, perceived necessity, and concerns patients have regarding their illness and prescribed medication [18]. Respondents indicate their degree of agreement with each individual statement about medicines on a 5‐point Likert scale. It is then possible to differentiate between patients on the basis of their beliefs about the necessity of their medication and their concerns about taking it. Patients can be classified into four different categories: accepting (high necessity and low concerns), ambivalent (high necessity and high concerns), skeptical (high concerns and low necessity), and indifferent (low concerns and low necessity) [32,33].

### Participant Timeline

**Figure 5:**
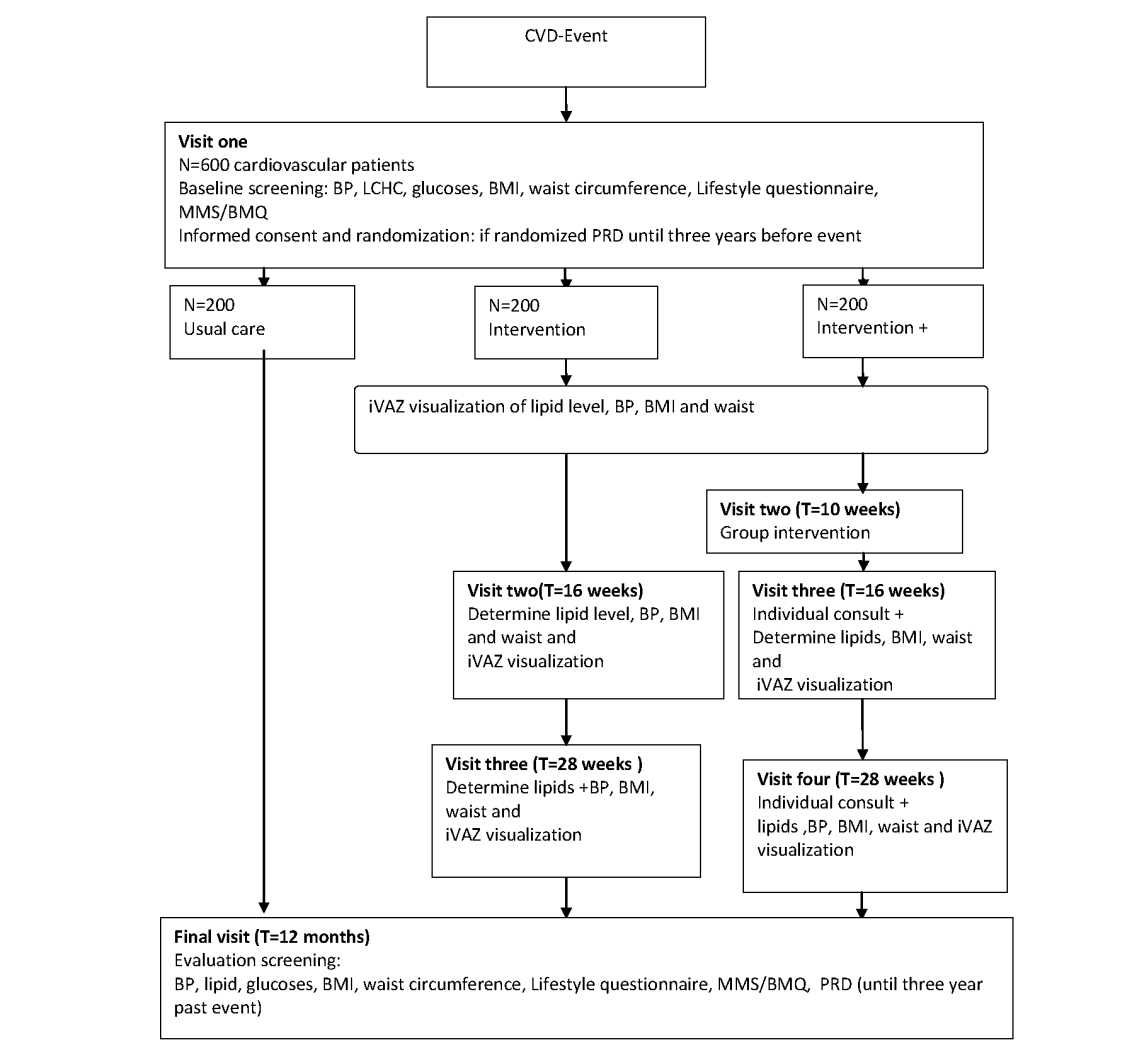
Patient flow chart.

Baseline scores will be collected for all groups. Follow-up scores will vary depending on group and will be collected at 6 and 12 weeks (all groups) and 16 and 28 weeks (intervention groups II and III) (see Figure 5 for flow chart).

### Sample Size

This study is mainly powered on the primary outcome, the detection of a significant difference between the three degrees of care (usual, additional website, additional counseling) on medication adherence as determined by refill records of computerized pharmacy systems. Based on previous research in our population and data from the literature [26], we estimate that the adherence at the start of the study will be 65% in each group with a standard deviation (SD) of 30%. We hypothesize that the intervention given in group II and the intervention given in group III will result in an increase of 10% in adherence to treatment, resulting in mean adherence rates of 75% and 85% in groups II and III, respectively. To detect these differences in medication adherence the estimated group size with a power of 80% and an alpha of .05 (2‐sided) would be 200 in each group, resulting in 600 participants in total.

### Recruitment

All cardiovascular patients who receive the regular cardiovascular preventive care will be asked to participate by a nurse when they arrive at the outpatient clinic for their screening consult. Patients will receive a letter explaining the study, documenting their ability to withdraw at any time without explanation, and confirming that their medical care will in no way be influenced by their decision regarding participation. At a minimum of 24 hours later, written consent will be sought by a research assistant prior to the patient entering the study.

We chose to include all cardiovascular patients in our study rather than only nonadhering patients as done in many other studies [6,9,34]. The reason is that we plan to do a 3-year follow-up and want to be able to see how adherence develops over time for initial adherers and nonadherers alike.

### Assignment of Interventions

Patients who meet the criteria and consent to participate will then be randomized by the nurse stratified by department (eg, neurology, vascular surgery, and cardiology) in a 1:1:1 ratio into one of the three groups using computer randomization.

### Blinding

The principal investigator and the researcher will be blind to randomization. However, due to the need for active participation, the patient, nurse, and pharmacist delivering the individual consultations will not be blind to assignment of individuals in group III.

### Data Collection and Management

The primary data collected will be provided by the initial screening. Obtained data from the screening are blood lipid levels, blood pressure, waist circumference, body mass index, blood glucose levels, and medication use. Lifestyle is evaluated through a questionnaire which is a compilation of existing validated questionnaires regarding demographic data, smoking, alcohol use, physical activity, and eating habits. For each of these lifestyle issues, patient’s motivation to change is evaluated [16]. Adherence is measured by the MMS [17] and beliefs about medication by the BMQ [18].

To monitor whether the website intervention is used, log‐in information per patient, expressed as the number of log‐ins and times and dates of log-in, will be recorded.

To measure the nurses’ performance skills required in the individual consultations, the behavior change counseling index will be used [35]. This validated checklist aims to measure the nurses’ competence in behavior change counseling and adaptation of motivational interviewing in healthcare settings. The group consultations are videorecorded and evaluated in order to validate the quality of the motivational interviewing techniques applied.

Data will be entered by the nurses who perform the screening and the intervention consults in iVAZ. iVAZ is a secured website which can only be entered by the participants by using their social security codes and by selected nurses using security codes. In addition, all patient pharmacists will receive a letter of information about the trial, consent of the ethical committee, and the informed consents of the participants. They will be asked to send the data on refill records of their computerized pharmacy systems through a secured email address. All the data will be anonymized according to the privacy protocols from the ethical committee and imported by the researcher into SPSS (IBM Corp).

### Statistical Methods

The data will be analyzed based on the intention‐to‐treat principle and evaluated using SPSS, with descriptive statistics (mean, median, SD, and interquartile range) being determined for all variables. The data will be presented in quantitative format (eg, biometrics, laboratory results, blood pressure, lifestyle scores, adherence score on the basis of refill data, and the MMS) and in descriptions of observed effects (eg, change in BMQ, determinants for adherence, evaluation of the use of iVAZ, and appreciation of nurse intervention).

To evaluate the difference between the groups, an analysis of variance test will be performed on the outcome measures for the three patient groups. The independent variable will be the three intervention groups. The dependent variable is medication adherence measured with the dedicated calculation of refill data. Specifically, we will compare the difference between the first and last time-point between groups for the primary and secondary outcomes measures. For the intervention groups II and III, we will also compare the outcomes of the clinical data at 16 and 28 weeks. To correct for multiple comparison, a Duncan’s multiple range test will be performed. Furthermore, we will perform a receiver operating characteristic (ROC) curve analysis to compare the outcome of the screening instruments (MMS and BMQ) with the pharmacy refill dates. In the ROC curve plot, specificity of the questionnaire is on the x-axis and sensitivity of the question is on the y-axis.

Plausible relations between parameters of cardiovascular risk factors, motivation to change, socioeconomic class, and parameters of adherence (calculated refill score and BMQ and MMS scores) will be tested in a univariate manner. Individual parameters will be tested for normality using the Kolomogorov-Smirnov test in order to select adequate univariate tests. Multiple logistic regression analysis will be performed to assess the relative importance of selected parameters for the likelihood of low adherence, as defined by the refill data algorithm. In all analyses, potential confounders will be included if they independently changed the beta coefficient for dedicated calculation of refill data by at least 5% or when consensus about inclusion existed within the team of researchers supported by clinical evidence from literature.

Missing data is unfortunately very common in eHealth research. We follow the recommendation for eHealth research to use the multiple imputation technique in SPSS when analyzing our dataset with missing observations [36].

### Ethics and Dissemination

The study protocol has been approved by the local ethical committee before inclusion of patients into the study. The study has been registered (trial registration ID number NCT01449695, approved May 2011). Subjects may leave the study protocol at any time for any reason without any consequences for regular cardiovascular care. The investigator or patient specialists may also decide to withdraw a subject from the study for urgent medical reasons.

## Discussion

Nonadherence to medication prescriptions in cardiovascular patients reduces the positive effects of medical treatment in chronic care. However, improvement of medication adherence in these patients is a serious challenge. Patient beliefs, perceptions, and management of medication, their illness (intentional nonadherence), and skills to integrate medication taking in their daily life (unintentional nonadherence) need to be addressed to make an intervention successful.

There is no one‐size‐fits‐all solution for nonadherence [9,34] nor does previous research provide evidence to choose a single intervention [37]. By reviewing the literature it becomes evident that determinants for nonadherent behavior are complex, and underlying theory for a successful intervention is frequently lacking [34,38,39]. In a review of 193 health behavior change articles, only 36% of the authors mentioned a theory and only 22% of them applied the theory [40].

We based our method on HBM, adopted the approach of Horne [41], and defined the main determinants of nonadherent behavior in intentional and nonintentional determinants. Because we address both types of determinants, we expected to develop an intervention that will be more successful than most existing interventions, which only take into account one of these sets of determinants.

Specifically, by choosing a group consultation, information is provided in an efficient manner and the patient is given an opportunity to discuss the need for adherence (intentional nonadherence) as well as getting practical information (unintentional adherence) with peers. Further tailoring the intervention in individual contacts provides the opportunity for the nurse to identify the need to change objectives of unintentional or intentional nonadherence (or a mix of both). These individual consultations are patient‐centered, with emphasis on patient perspectives and shared decision making [42]. The individual website and visualization of personal cardiovascular risk furthermore addresses one of the difficulties in cardiovascular adherence: awareness of the influence of taking medication on personal cardiovascular risk [40]. Lastly, the combination of Web-based intervention with face-to-face contact is expected to give better results than either alone [43]. Based on this integration of factors, we hope that the resulting data of this trial will contribute important knowledge about adherence in this population.

## Abbreviations

BMQ: Beliefs about Medication Questionnaire
CVD: cardiovascular disease
HBM: health belief model
iVAZ: Interactive Dossier Vaatzorg
MMS: Modified Morisky Scale
SD: standard deviation
